# Synthesis, Characterization, and Performance of Semi-Refined Kappa Carrageenan-Based Film Incorporating Cassava Starch

**DOI:** 10.3390/membranes13010100

**Published:** 2023-01-12

**Authors:** Camellia Panatarani, Danar Praseptiangga, Putut Ismu Widjanarko, Sundoro Yoga Azhary, Puspita Nurlilasari, Emma Rochima, I Made Joni

**Affiliations:** 1 Department of Physics, Faculty of Mathematics and Natural Sciences, Universitas Padjadjaran, Jl. Raya Bandung-Sumedang KM 21, Jatinangor 45363, West Java, Indonesia; 2 Functional Nano Powder University Center of Excellence, Universitas Padjadjaran, Jl. Raya Bandung-Sumedang KM 21, Jatinangor 45363, West Java, Indonesia; 3 Research Collaboration Center for Marine Biomaterials, Jl. Raya Bandung-Sumedang KM 21, Jatinangor 45363, West Java, Indonesia; 4 Department of Food Science and Technology, Faculty of Agriculture, Universitas Sebelas Maret (UNS), Jl. Ir. Sutami 36 A, Jebres 57126, Central Java, Indonesia; 5 Department of Fishery Processing Technology, Faculty of Fishery and Marine Science, Universitas Padjadjaran, Jl. Raya Bandung-Sumedang KM 21, Jatinangor 45363, West Java, Indonesia; 6 Department of Agro-Industrial Technology, Faculty of Agro-Industrial Technology, Universitas Padjadjaran, Jl. Raya Bandung-Sumedang KM 21, Jatinangor 45363, West Java, Indonesia

**Keywords:** cassava starch, semi-refined kappa carrageenan, shelf life, active food packaging

## Abstract

This paper reports the incorporation of cassava starch (CS) at various concentrations into a previously developed ZnO/SiO_2_-semi-refined kappa carrageenan-based film (SRκC) bionanocomposite and evaluates its performance as minced chicken edible packaging. The incorporation of CS into SRκC-based films aims to provide multifunctional food packaging with enhanced surface morphology, thickness, mechanical properties, and transparency. The effect of the incorporation of various mixing ratios of CS and SRκC (CS:SRκC ratios of 1:3, 1:1, and 3:1) was investigated. The results show that the surface morphology, thickness, and mechanical properties of the SRκC-based films are increased by incorporating CS. Interestingly, a significant shelf-life improvement of up to 6 days is obtained for the application of the CS:SRκC 1:3 film as minced chicken packaging. It is concluded that the incorporation of CS into SRκC-based film is promising for extending the shelf life of minced chicken samples.

## 1. Introduction

Minced chicken is widely consumed by people due to its low-fat, high-protein, and easy-to-cook properties [1]. However, minced chicken is susceptible to microbial contamination, lipid oxidation, spoilage, and discoloration [2]. Consequently, the quality of minced chicken gradually degrades and tends to become waste during sorting. This has become an enormous problem in the supply chain of minced chicken. To overcome this problem, minced chicken is usually packed with synthetic polymer (plastic) during refrigeration [3]. However, food packaging using synthetic polymer generates a global environmental concern. Therefore, instead of synthetic polymer, a biopolymer material is the promising alternative for environmentally friendly food packaging.

Biopolymer material resources, such as seaweed, are abundant in Indonesia and many other places worldwide. The application of biopolymer material from seaweed as food packaging material offers several benefits due to its availability, smaller impact on the food chain, and relative safety from chemical exposure and fertilizers [4]. Among the several biopolymer materials derived from seaweed, semi-refined kappa carrageenan-based film (SRκC) is an interesting candidate for utilization as edible biodegradable food packaging (biopackaging) [5]. Compared to other types of carrageenan, SRκC has the strongest gelling ability and is naturally hydrophobic to water resistance due to its low sulfate substitution content [5,6]. Moreover, SRκC is cheap in terms of production costs compared to refined carrageenan. However, the use of SRκC-based film as biopackaging material is limited due to its water vapor permeability and brittleness [7]. To overcome this weakness, various nanoparticles (NPs) are incorporated as nanofillers into SRκC-based films to enhance their film characteristics and packaging properties [8,9,10,11,12,13]. The incorporation of ZnO and SiO_2_ NPs enhances the functionality of antimicrobial activity against numerous microorganisms and mechanical stability, respectively [14]. However, the incorporation of ZnO and SiO_2_ NPs into SRκC-based films tends to reduce film transparency and, without a proper surfactant, may cause a reduction in their mechanical properties [10]. This weakness is probably because NPs are easily agglomerated and unstable in aqueous suspension [11]. NP agglomeration in suspension can diminish the formation of the desired film properties [15]. In order to break up NP agglomerates in suspension, the bead-milling method was utilized in our previous studies [11,15,16,17,18]. By using the bead-milling method, well-dispersed NP suspensions and the expected surface functional groups could be achieved [9,13]. Furthermore, we found that the best formula for NP incorporation into carrageenan-based film by the bead-milling method is SiO*_2_*–ZnO 1:3 [12]. Nevertheless, the incorporation of SiO_2_–ZnO 1:3 into carrageenan-based film tends to produce rough surface morphology and a thick film. Therefore, it is important to modify the film matrix by adding the proper type of starch into carrageenan-based film.

Previously, we investigated the reinforcement of carrageenan/starch-based film by the bead-milling method, which successfully improved the tensile strength properties and thermal degradation of the film [19]. Film transparency is important for customer preferences to assess food freshness and control food nutrient loss; however, the incorporation of corn starch into carrageenan-based film tends to reduce film transparency [19]. Cassava starch (CS) is a biopolymer that is well known to enhance the mechanical properties of composites due to its hydrophilic and chemical structure [20]. In addition, CS is cheap, abundant in nature, and exhibits high viscosity properties [20]. Thus, it is important to investigate CS incorporation into SRκC-based film to resolve the above drawback.

The present study aims to incorporate cassava starch into SRκC-based film with the best composition ratio of SiO_2_–ZnO nanofillers of 1:3. The effect of various mixing ratios of CS and SRκC was investigated in detail in terms of their performances on water vapor pressure, optical properties, mechanical properties, and antimicrobial activity against both *E. coli* and *S. aureus*. Furthermore, the best ratio of CS and SRκC matrix film was applied as minced chicken packaging, and its performance was investigated based on several food quality parameters, such as pH, total volatile base nitrogen (TVBN), total plate count (TPC), and thiobarbituric acid-reactive substance (TBARS).

## 2. Materials and Methods

### 2.1. Materials

Semi-refined kappa carrageenan (SRκC) powder was purchased from Galic Artabahari, Co., Ltd. (Bekasi, Indonesia). Cassava starch (CS) was purchased from Budi Starch & Sweetener, Co., Ltd. (Lampung, Indonesia). SiO_2_ nanoparticle (NP) powders and ZnO NP powders with particle sizes of ±60 nm and ±160 nm, respectively, were received from JP Cipta Nanotech Indonesia Ltd. (Bandung, Indonesia). Hydrochloric acid (HCl) 37% (analytical reagent) was purchased from PT. Smart Lab Indonesia (Banten, Indonesia). Sodium dodecyl sulfate (C_12_H_25_O_4_SNa) (SDS) FCC was purchased from PT. Elo Karsa (Bandung, Indonesia). Bacterial cultures of *Escherichia coli* (*E. coli* FNCC 0091) and *Staphylococcus aureus* (*S. aureus* FNCC 0047) were obtained from the Department of Biology, Universitas Padjadjaran (Bandung, Indonesia). Nutrient agar, nutrient broth, plate count agar, glacial acetic acid, and sodium chloride (NaCl) were purchased from Merck, Co. (Darmstadt, Germany). Malondialdehyde tetrabutylammonium salt (MDA salt) and thiobarbituric acid (TBA) reagent were purchased from PT. Elo Karsa (Bandung, Indonesia). The minced chicken sample was purchased from PT. Lion Superindo, Indonesia.

### 2.2. Preparation of Films at Various Cassava Starch (CS) Concentrations

**Figure 1:**
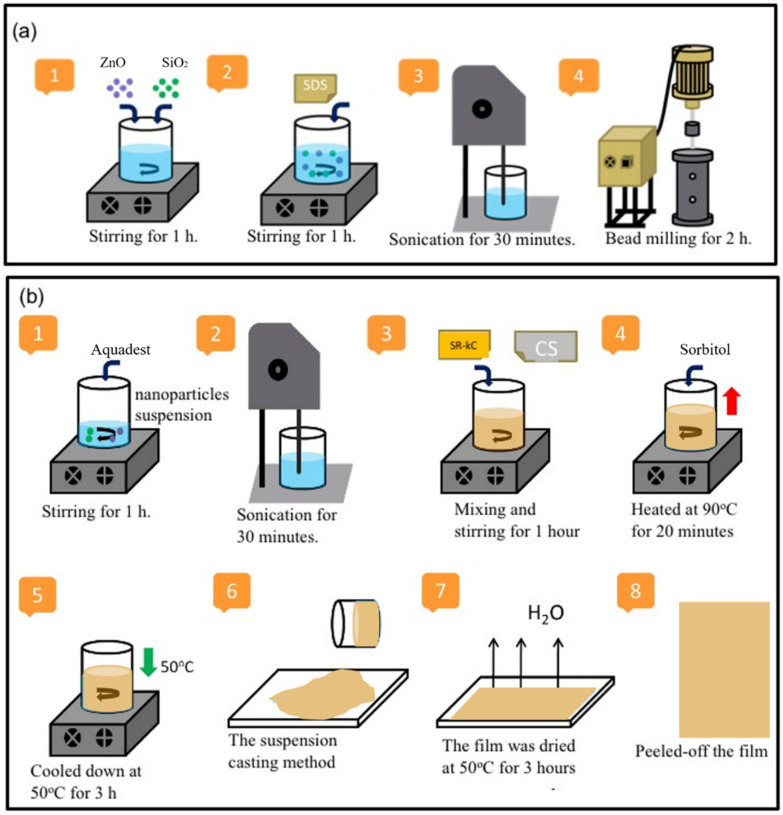
Schematic illustration of (**a**) preparation of ZnO−SiO_2_ nanoparticle suspension; (**b**) incorporation of CS at various concentrations into previously developed ZnO/SiO_2_−SRκC bionanocomposite film.

The ratio of ZnO and SiO_2_ NP fillers in the composite film was 3:1 according to previous publications [12]. A mixture of ZnO and SiO_2_ NP suspension was prepared using the bead-milling method. ZnO and SiO_2_ NP, correspondingly, 0.3 and 0.1 g, were mixed into 97.0 g of aquadest and stirred for 1 h. Then, 0.04 g of sodium dodecyl sulfate (SDS) FCC, which is commonly used as a safe food emulsifying agent, was added into the mixture under stirring conditions for 1 h and sonicated for 30 min to prepare the NP suspension. The NP dispersion was prepared by the bead-milling process in a vessel of 150 mL containing zirconia beads (80 µm in size) for 2 h to generate a good dispersion of nanoparticles in the suspensions, as shown in Figure 1a. A sample of the ZnO−SiO_2_ NP suspension was measured using Horiba Nanoparticle Analyzer SZ 100 series instruments for particle size distribution and zeta potential.

Figure 1b shows a schematic representation of the bionanocomposite film preparation. A 15 g amount of as-prepared ZnO−SiO_2_ NP suspension was subsequently added into 96.6 g of aquadest and sonicated for 30 min. Various mixtures of CS and SRκC solution (with CS:SRκC ratios of 1:3, 1:1, and 3:1) were mixed into the ZnO−SiO_2_ NP suspension and stirred for 1 h. This mixture was blended with 1 g of sorbitol and 0.04 g of SDS and heated at 90°C under stirring conditions for 20 min. Once the temperature had decreased to 50 °C, the film solution was poured onto a plastic plate (24 cm × 16 cm) until it turned into a gel before drying it in an oven (50 °C) for 3 h. The other films from various CS:SRκC ratios were produced with a similar method, except for the SRκC control film.

The SRκC and SRκC–NP control films were prepared as separate films to evaluate the developed film’s performance. The SRκC and SRκC–NP films were prepared correspondingly as film containing only SRκC and with SiO_2_–ZnO NP incorporation, as shown in Table 1. The bionanocomposite films were prepared using the solution casting method as explained in our previous report with minor modifications to the heating temperature for the SRκC control film and SRκC–NP film [10]. The SRκC control film was prepared by dissolving 2 g of SRκC and 1 g of sorbitol into 97 g of aquadest and heating at 70 °C under stirring conditions for 30 min. Finally, the dried film was peeled off from the plate and stored in a relatively low RH cabinet for further characterization.

### 2.3. Characterization of Bionanocomposite Film

#### 2.3.1. Fourier-Transform Infrared (FTIR)

The characteristics of functional groups of the bionanocomposite films were determined using FTIR spectrometry (ATR-FTIR Thermo Nicolet iS5). The FTIR spectrum was measured at the wavenumber range of 4000–400 cm^−1^ at room temperature [21].

#### 2.3.2. Optical Properties

The optical properties (transparency and UV screening) of the bionanocomposite films were determined by using a UV–VIS spectrophotometer (Shimadzu, UV-1800). The film sample (3 cm × 1 cm) was placed in a thin cuvette for UV–VIS characterization [22]. The UV–VIS spectrophotometer was measured in the wavelength range of 200–700 nm. The UV barrier properties and transparency of the film were evaluated by measuring percent transmittance at 280 nm and 550 nm, respectively.

#### 2.3.3. Surface Morphology

The surface morphology of the bionanocomposite films was determined by scanning electron microscopy (SEM) and atomic force microscopy (AFM) [23]. The SEM analysis was conducted using the Hitachi Model SU3500 instrument, while the AFM analysis was conducted using the Keysight 5500 instrument (N9410S, Keysight Technologies Canada Inc., Ottawa, ON, Canada). For the AFM analysis, images of the film samples (256 × 256 pixels) were acquired by scanning square areas of 5 × 5 μm with a scanning speed of 0.3 line/s.

#### 2.3.4. Wettability

The wettability of the bionanocomposite films, such as water contact angle (WCA) and critical surface tension (CST), was utilized by using ImageJ software. The WCA was determined based on a drop of distilled water (∼10 μL) onto the surface of the film using a microsyringe pump. The CST was determined based on the sessile drop technique using three different probing liquids, such as deionized water, formamide, and n-hexadecane [24]. The surface tension (γ) of each liquid test was entered as the *x*-axis, and the cosine value from the contact angle (θ) was entered as the *y*-axis in the linear regression analysis.

#### 2.3.5. Thickness

The thickness was measured at five different points of the bionanocomposite film. The thickness measurement was determined using a digital micrometer (KRISBOW KW06-85) with a precision of 0.001 mm [25]. 

#### 2.3.6. Mechanical Properties

The mechanical properties of the bionanocomposite films, such as tensile strength (TS) and % elongation at break (EB) were evaluated using the Zwick Type 0.5 universal testing machine. According to ASTM method D 882-02, the bionanocomposite films were cut into rectangular strips (2.5 cm wide and 15 cm length) by using a precision double-blade cutter (Model LB.02/A, Metrotech, SA, San Sebastian, Spain) [26]. The tensile properties were measured by operating the machine in the tensile mode with an initial grip separation of 50 mm and a crosshead speed of 50 mm/min.

#### 2.3.7. Antimicrobial Activity

The antimicrobial activity of the bionanocomposite films against two foodborne pathogenic bacteria that are generally present in contaminated minced chicken products, *E. coli* (Gram-negative) and *S. aureus* (Gram-positive), was evaluated by examining the inhibition zone on solid media, according to the agar diffusion method [27]. The two foodborne pathogenic bacteria *E. coli* and *S. aureus* were selected because they own different cell wall structures. The cell wall structure of *E. coli* is a complex layer with a fine peptidoglycan layer, while the cell wall structure of *S. aureus* is thick with many layers of peptidoglycan surrounded by an outer membrane [28]. The bacterial strains were grown in nutrient broth and incubated for 1 day at 37 °C, and the cell concentrations were adjusted to 0.5 McFarland units containing 1.5 × 10^8^ CFU/mL. The films were cut into a square shape (1 cm × 1 cm) and placed on a Petri dish containing nutrient agar inoculated with 0.1 mL of inoculum having 10^8^ CFU/mL *E. coli* and *S. aureus*. Furthermore, the Petri dish was incubated for 1 day at 37 °C, and the inhibition zone surrounding the films was measured in mm using a digital micrometer. All experiments were carried out in triplicate.

#### 2.3.8. Water Vapor Permeability (WVP)

The WVP of the bionanocomposite films was determined gravimetrically according to the ASTM E 96-00 with slight modification [21]. For this purpose, 3 g of anhydrous CaCl_2_ was filled in a test cup. Then, the cup was sealed with melted paraffin around the edges and stored in a humidity chamber at 25 °C (60% RH). The cup was weighed and recorded periodically every 1 h for 7 h. The weight changes were recorded as a function of time. The WVP (10^−6^ g·h^−1^·m^−1^·Pa^−1^) was derived from the slope (g·h^−1^) of the linear regression.

#### 2.3.9. Degradability

The degradability of the bionanocomposite films was determined by the soil burial test, which evaluates the sample’s weight loss over time [29]. For this purpose, the bionanocomposite film (3 cm × 3 cm) was buried in a cup filled with soil. The depth of the soil in the cup was 10 cm. To maintain the moisture inside the cup, the cup was covered and placed under a temperature of 25 °C with 60% relative humidity for 28 days of observation. During the weight loss observation, the sample was weighed periodically every 7 days. The degradation rate was determined by the sample’s weight loss over time.

### 2.4. Performance of Minced Chicken Edible Packaging

#### 2.4.1. Packaging of Minced Chicken Sample

The test cup was used for minced chicken packaging. The test cup was filled with 50 g of minced chicken meat and covered with the respective bionanocomposite films under aseptic conditions. Then, the packaged minced chicken sample was placed on the plate and stored at refrigeration temperature for 12 days. During observation, the sample was investigated periodically every 3 days. The film packaging performance measurements were pH, weight loss (WL), total volatile base nitrogen (TVBN), total plate count (TPC), and thiobarbituric acid-reactive substance (TBARS), which were employed as indicators of chicken degradation, microbiological quality, and malonaldehyde concentration in minced chicken, respectively.

#### 2.4.2. pH Value of Packaging Sample

The pH of a minced chicken sample is a measurement of acidity. Minced chicken pH is one of the main technical attributes that drive customers’ consideration of minced chicken quality. In addition, the pH of minced chicken affects eating quality characteristics such as juiciness, tenderness, and taste. Thus, the pH of the sample was determined using the Starter 3000 pH meter (Ohaus Co., Zürich, Switzerland).

#### 2.4.3. Total Volatile Base Nitrogen (TVBN)

Total volatile basic nitrogen (TVBN) is one of the methods to reveal the freshness degree of minced chicken, by analyzing the presence of nitrogenous compound quantity in the sample. The TVBN of the minced chicken sample was measured according to Chinese standards (GB 5009.228-2016) and previous literature [30]. Briefly, 10 g of minced chicken sample was transferred to a Kjeldahl distillation unit. The volatile biogenic amines were distilled, and the distillate was collected with 10 mL of boric acid solution (20 g/L) containing 5 droplets of mixed indicator that was made by dissolving 0.2 g of methyl red and titrated with 0.01 M HCl solution. Thus, the TVBN content was calculated according to the amount of HCl used during titration and expressed as mg/100 g.

#### 2.4.4. Total Plate Count (TPC)

Total plate count (TPC) is one of the methods to estimate the total number of microorganisms in minced chicken. The TPC was determined using the pour plate method. Briefly, 10 g of the minced chicken sample was homogenized in 90 mL of sterile 85% NaCl solution with a blender (HBM-400B, HBM Biomed, Tianjin, China) at room temperature. An appropriate dilution was serially prepared. Then, 1 mL of each dilution was spread onto plate count agar media (Merck, Germany). The prepared plates were incubated at 37 °C for 7 days for TPC. Thus, all counts were expressed as log colony-forming unit (CFU)/g, and all measurement was conducted in triplicate.

#### 2.4.5. Thiobarbituric Acid-Reactive Substance (TBARS)

Thiobarbituric acid-reactive substance (TBARS) is one of the methods to detect lipid oxidation in minced chicken. Briefly, 10 g of the minced chicken was mixed with 20 mL of trichloroacetic (TCA) 7.5% (*w*/*v*) and agitated for 1 h to extract the MDA. Then, 5 mL of the filtrate was combined with 5 mL of 0.02 M 2-thiobarbituric acid (TBA) and heated at 95 °C for 30 min in a water bath. The absorbance of the sample was measured at 530 nm via a UV–VIS spectrophotometer. The calibration curve using known concentrations of MDA was used to calculate the TBARS index. The results were expressed as mg of MDA/kg of minced chicken sample.

#### 2.4.6. Weight Loss (WL)

The weight loss of the film sample was determined by measuring the film weight before and after degradation over time [31,32]. 

### 2.5. Statistical Analysis

Statistical analysis of the data was performed by one-way analysis of variance (ANOVA) using the SPSS statistical program (SPSS 16.0 program for Windows, SPSS Inc., IBM, Chicago, IL, USA). The difference between the mean value of the results was compared using Duncan’s multiple range test at the 0.05 level of significance. The results data were presented as the mean ± SD (standard deviation), and *p* < 0.05 was taken as the minimum level of significance.

## 3. Results and Discussion

### 3.1. Particle Size Distribution and Zeta Potential

**Figure 2:**
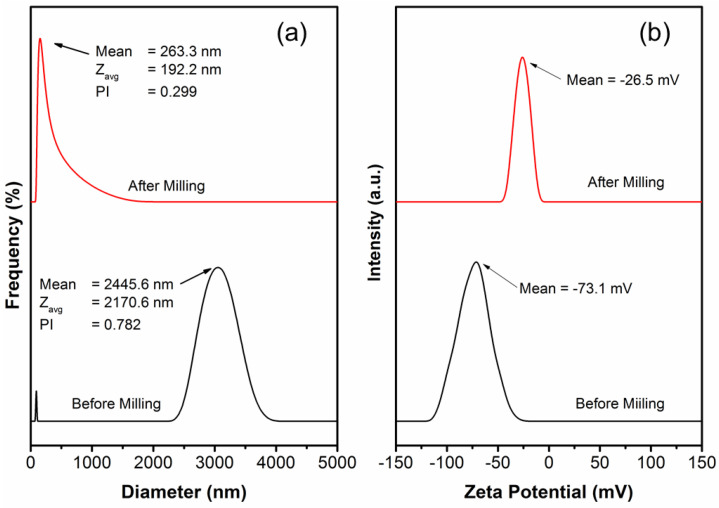
ZnO−SiO_2_ NP suspension before and after bead milling: (**a**) particle size distribution and (**b**) zeta potential.

The particle size distribution and zeta potential are shown in Figure 2. The average particle size of the ZnO−SiO_2_ NP suspensions before and after the bead-milling process are 2445.6 nm and 263.3 nm, respectively. Although the dispersion stability of the ZnO−SiO_2_ NP suspensions before the bead-milling process is very high (−73.1 mV), the stability occurs at a higher size distribution due to agglomeration. In contrast, the size distribution of the ZnO−SiO_2_ NP suspension after the milling process shows a single peak with a low polydispersed index (0.299). This indicates a monodispersed suspension with relatively stable dispersion (−26.5 mV). The effectiveness of the milling process of the ZnO−SiO_2_ NP suspensions may be due to one of the particles acting as a bead to enhance particle collision. This result designates that the bead-milling process successfully reduces the particle size distribution, and a proper dispersing agent enables the generation of a stable dispersion.

### 3.2. Fourier-Transform Infrared (FTIR)

**Figure 3:**
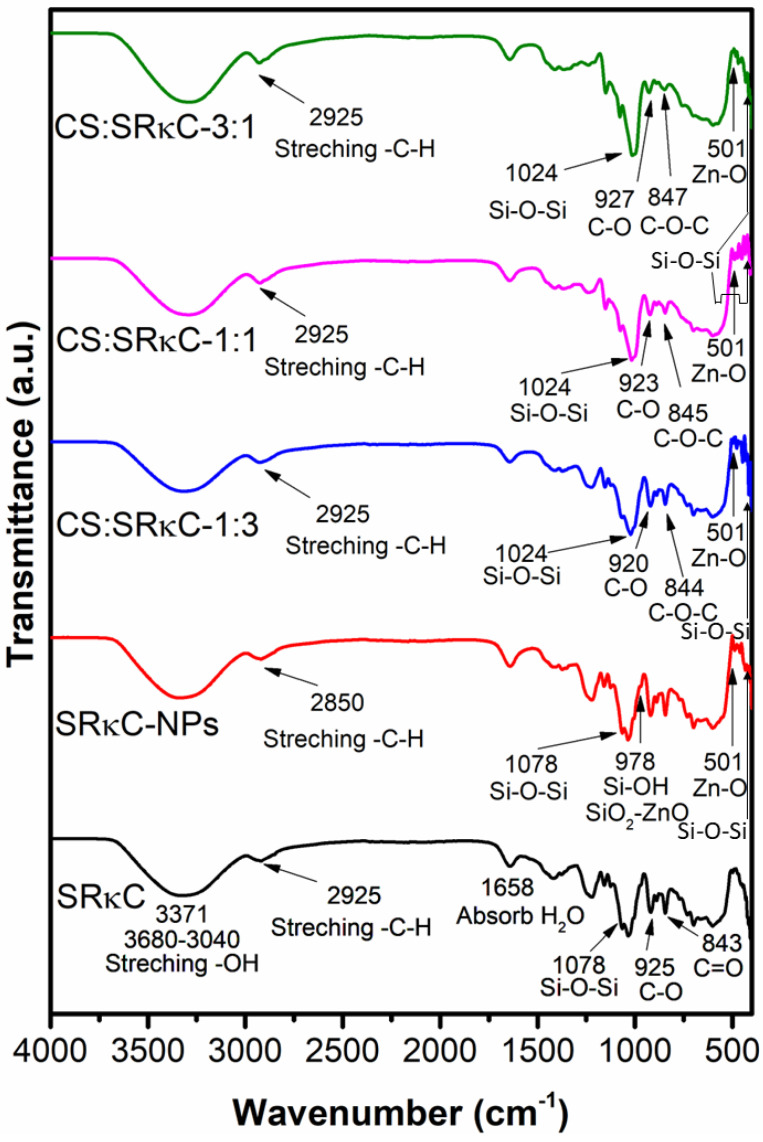
FTIR spectra of bionanocomposite films at various ratios of Cs:SRκC compared to the control film.

The chemical groups of the SRκC-based films at the molecular level are usually observed by FTIR spectra, as shown in Figure 3. The FTIR spectra of the SRκC film appear at 3371 and 1658 cm^−1^ corresponding to the hydroxyl (O−H) stretching of hydrogen bonds. The presence of the hydroxyl group is probably obtained from the SRκC chemical structures and adsorbed water [13,33]. The other band observed at 2925 cm^−1^ corresponds to C−H asymmetric stretching in the alkaline groups of the absorbed water [21]. The FTIR spectra of the SRκC−NP films show additional bands at 430–520, 501, and 473 cm^−1^. The band at 430–520 and 501 cm^−1^ corresponds to the Zn-O band, demonstrating the presence of zinc oxide [26], while the band at 473 cm^−1^ corresponds to the intense Si-O-Si band, demonstrating the presence of SiO_2_ [22,23]. Therefore, the FTIR of the SRκC−NP films indicates the presence of the ZnO−SiO_2_ NP vibration group [13]. This result demonstrates that the ZnO−SiO_2_ NP filler is well incorporated into the SRκC-based films. The spectra of the SRκC and SRκC−NP films are subsequently used as a control for the other films under investigation.

The FTIR spectra of the SRκC−NP film at various CS concentrations (SRκC-CS) are also presented in Figure 3 and compared with the control films. In contrast to the control, additional bands at 844–847, 920–923, and 1080–1180 cm^−1^ appear. Bands at 1080–1180 cm^−1^ corresponding to the C-O band from the typical CS structure are observed [21]. Additional bands at 844–847 and 920–923 cm^−1^ are detected, corresponding to the C-O-C band and the 3,6-anhydro-D-galactose group of the CS and SRκC [22]. This result demonstrates that CS is well incorporated into the SRκC films. There is no significant shifting of the position of the peaks for the C-H, C-O-C, Si-O-Si, and Zn-O vibrations in the SRκC-CS film at various concentrations. This result indicates the enhancement of the intermolecular interaction between NP filler due to the additional CS. Thus, the additional CS improved the NP filler and SRκC matrix network formation.

### 3.3. Surface Morphology

**Figure 4:**
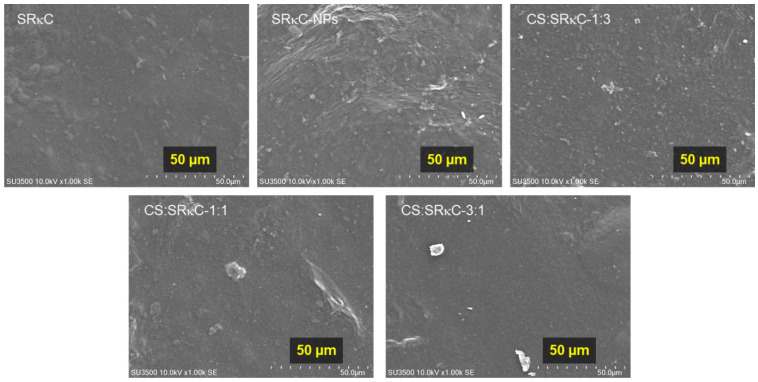
SEM images of SRκC films at various CS concentrations.

**Figure 5:**
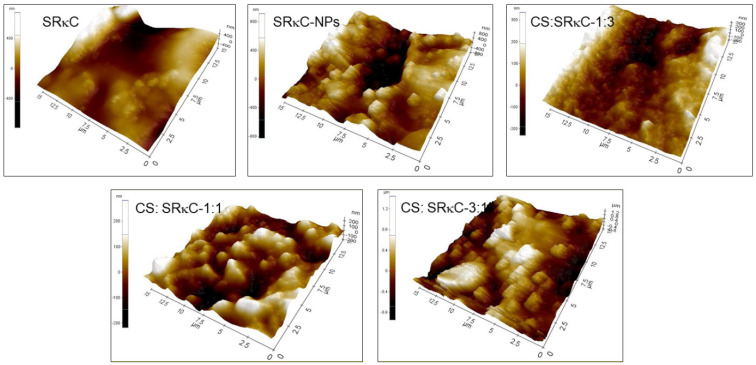
AFM images of SRκC films at various CS concentrations.

The surface structures of all films based on SEM and AFM observations are shown in Figure 4 and Figure 5, respectively. The SEM images in Figure 4 show the incorporation of ZnO−SiO_2_ NPs into SRκC causes a rougher surface compared to the SRκC control film. In addition, the SRκC−NP film shows a nonhomogeneous surface structure with a cracked surface. This may be due to the presence of NPs filling the empty spaces in the macromolecular structures of SRκC, consequently forming the rough surface morphology of the SRκC−NP films. This phenomenon also occurs when nanoparticles are included as filler in iota carrageenan-based film, as reported in our previous work [34]. The rough surface structure of the SRκC−NP film may increase the water vapor barrier properties of the film [22]. Therefore, additional CS is essential to improve the surface properties of the film.

Remarkably, the surface structure of the CS:SRκC 1:3 film becomes a more homogeneous structure than the SRκC−NP film. In comparison, the CS:SRκC 1:1 film shows a smoother surface structure morphology than CS:SRκC 1:3. The more CS added into the SRκC-based film, the smoother the surface structure of the film obtained (CS:SRκC 3:1). This is in agreement with previous studies by Tongdeesoontorn et al. (2012), who showed that the internal structure of gelatin-based film is increased following the incorporation of CS [35]. 

AFM was employed to obtain the contrast of the film’s surface roughness, as shown in Figure 5. The surface of the SRκC−NP film shows a rougher surface contour than the SRκC film. The rough surface morphology of the SRκC−NP film is because of the presence of ZnO−SiO_2_ NPs. In contrast, the incorporation of CS in the SRκC−NP film at various CS concentrations shows a smoother surface contour than the SRκC−NP film. Moreover, the surface contour of the CS:SRκC 3:1 films with more CS incorporation shows the smoothest surface contour. The obtained AFM images are consistent with the SEM images, in which smoother surface morphology is obtained by adding more CS into the SRκC-based film [36]. This result demonstrates that the surface smoothness of the films is strongly affected by the presence of CS incorporation.

### 3.4. Wettability

**Figure 6:**
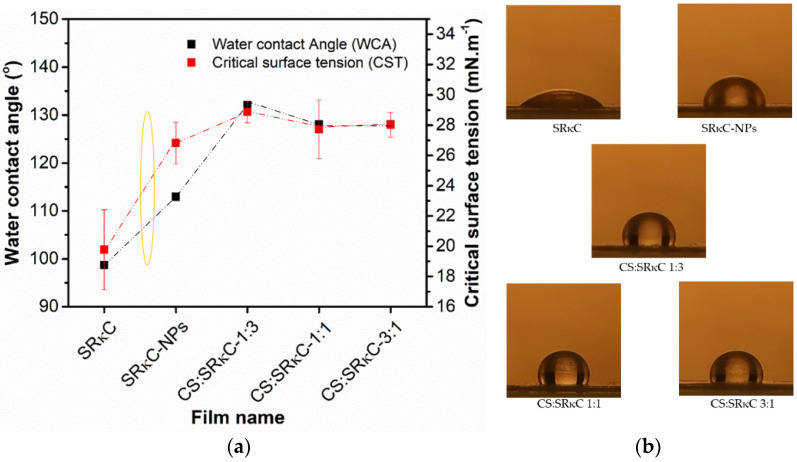
(**a**) Water contact angle and critical surface tension of bionanocomposite films; (**b**) images of water droplets on the surface of bionanocomposite films.

The wettability properties are shown in the form of water contact angle (WCA) and critical surface tension (CST) (Figure 6a). The CST of all the films is in the range of 18–22 mN/m, which is lower than the CST of water (72 mN/m). Thus, the WCA of all films is higher than 90°, indicating hydrophobicity characteristics [37]. The hydrophobicity characteristics of the films are also caused by nanoparticles and hydroxyl groups’ bonding interaction from water and plasticizer [38]. The hydrophobicity characteristics of the film surface offer several benefits to the film’s application in food packaging, such as the film’s ability to reduce water absorption and water penetration through its surfaces [39,40]. The incorporation of less CS concentration in the CS:SRκC 1:3 film exhibits the highest WCA and CST value than the other films. As shown in Figure 6a,b, the WCA value (133°) of the CS:SRκC 1:3 film is higher than the SRκC control film (98°) as the water drop spreads on the film surface. The improvement of the WCA to 35° (from 98° to 133°) is considered significant due to the surface transformation from hydrophobic close to the superhydrophobic criteria. The surface is hydrophilic when its WCA is less than 90°, and it is hydrophobic when its WCA is >90°. The driving force for switching from hydrophilicity to hydrophobicity is the high surface tension of water [41]. Thus, it is defined that the surface of the developed film is hydrophobic. However, the film with a higher CS concentration produces a lower WCA value. The film with a lower CS concentration (CS:SRκC 1:3) causes the surface to be rougher compared to the other two films (CS:SRκC 1:1 and CS:SRκC 3:1). This roughness may be responsible for the higher WCA value, consequently preventing the liquid from wetting the film’s surface [42]. 

### 3.5. Thickness

As shown in Table 2, the thickness of the SRκC film is 57.30 ± 8.17 ^a^ µm. The thickness of the SRκC−NP film does not show a significant increase (58.14 ± 7.85 ^a^) µm. In contrast, the film thickness of the CS:SRκC 1:3 is 55.70 ± 6.21 ^ab^ µm, and CS:SRκC 1:1 is 55.10 ± 8.07 ^ab^ µm, which are thinner than the SRκC−NP film. In contrast, the film with the highest CS concentration of 53.52 ± 7.33 ^b^ µm is CS:SRκC 3:1, which is the thinnest film compared to the others. Thus, the more CS added into the SRκC based film, the thinner the film obtained. The thickness of the CS:SRκC 3:1 film shows a significant decrease compared to the SRκC and SRκC−NP films due to the presence of CS. Thus, the more CS added into the SRκC-based film, the thinner the film obtained. The thinner film with CS addition due to the presence of amylose content in CS gradually gelatinized, consequently causing more water bound in the film matrix and reducing the film thickness [37,43,44]. Thus, the higher concentration of additional CS forms a thinner film, consequently affecting the optical properties, transparency, and opacity of the film.

### 3.6. Optical Properties

**Figure 7:**
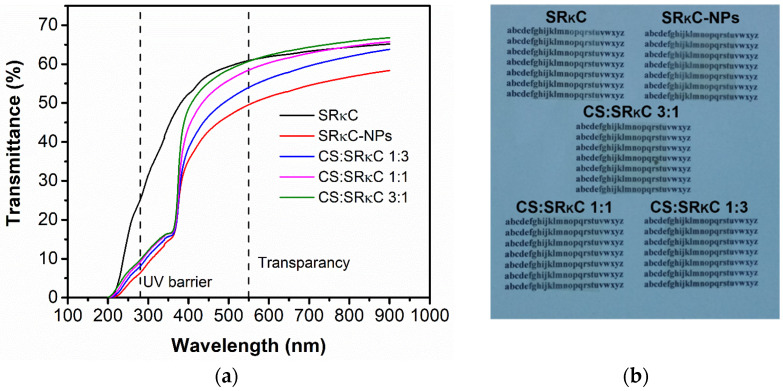
(**a**) UV–VIS spectra of CSRkC film with various CS concentrations in comparison with SRκC and SRκC−NP films; (**b**) pictures of respective bionanocomposite films.

Figure 7 shows the UV–VIS spectra of the SRκC films with various CS concentrations in comparison with the SRκC and SRκC−NP films. Two different UV–VIS responses are classified to evaluate the UV barrier at the wavenumber of 200–400 nm and transparency at the wavenumber of 400–700 nm. These are important properties of films related to the prevention of food oxidation caused by UV light, while transparency is related to customer preference. The SRκC film shows high transparency, but no UV barrier property is observed. In contrast, there is the presence of SiO_2_ and ZnO SRκC.

The transmittance spectrum of transparency (Table 3) for the SRκC film is 62.69%. The transparency of the SRκC−NP film reduces to 53.33% due to the presence of NPs in the film. If compared to the SRκC−NP film, the transparency of the SRκC−NP film at various CS concentrations is increased as more CS concentrations are added. Thus, the highest transparency is obtained from the CS:SRκC 3:1 film. In contrast, the presence of ZnO NP in the film leads to an increase in the UV barrier properties (Table 3) compared to the SRκC film, and this is also observed in the SRκC−NP film at various CS concentrations [45,46]. However, there is no significant difference in UV barrier properties in the SRκC−NP film at various CS concentrations. Therefore, the additional CS slightly affects the UV barrier properties. The transparency of the CS:SRκC 3:1 film at a higher CS concentration is close to the SRκC control film. However, it is important to investigate whether a higher concentration of CS in the film enhances mechanical properties.

### 3.7. Mechanical Properties

Table 2 shows the mechanical properties of the film. Mechanical properties are important characteristics of films to maintain the integrity of food packaging. The incorporation of less CS concentration in the SRκC (CS:SRκC 1:3) films causes stronger tensile strength (TS). In contrast, the TS reduces significantly with more addition of CS concentration: CS:SRκC 1:1 and CS:SRκC 3:1. Thus, a proper composition ratio of the CS:SRκC 1:3 films reinforced the adhesion force and subsequently enhanced the obtained TS. It is well known theoretically that the TS improves due to strong van der Waals forces in the presence of less CS, consequently strengthening the intermolecular forces between the NP, CS, and SRκC matrix [13,14]. 

The elongation of all films at various CS concentrations is significantly different compared to the control SRκC film (Table 2). CS:SRκC 1:1 shows higher elongation than the CS:SRκC 1:3 films. Moreover, the higher additional CS in the CS:SRκC 3:1 film shows higher elongation because the amylose content of the CS increases the flexibility of the molecular movements of the polymer chain interaction between the CS, NP, and SRκC matrix [25,37,38,41,44,47]. Consequently, it contributed to the film’s flexibility and good swelling ability. Hence, the more CS added improved the TS of the SRκC film; however, lower elongation properties were obtained.

### 3.8. Water Vapor Permeability (WVP)

The WVP of packaging film indicates its inhibition of water absorption from the environment to maintain food quality. As shown in Table 2, the WVP of the SRκC film was 1.05 ± 0.05 g·h^−1^·m^−1^·Pa^−1^. Then, the WVP value of the SRκC−NP film of 0.88 ± 0.11 g·h^−1^·m^−1^·Pa^−1^ decreased with the presence of NPs. Compared to the SRκC and SRκC−NP control, the WVP value of the SRκC−NP film at various CS concentrations was decreased because of the smooth surface structure of the film with CS incorporation. In accordance with the decreased WVP value of the SRκC−NP film at various CS concentrations, CS incorporation into bionanocomposite film reduced the biodegradable process. This phenomenon was because the microorganism infiltration into the film surface was inhibited. Thus, the full degradation process of the film slowly occurred. The decreased WVP by CS incorporation tended to decrease the film’s moisture barrier properties due to the slow water diffusion rate of water vapor molecules passing through the film in the presence of CS and NPs [1,41,48]. In addition, the slow water diffusion rate of water vapor molecules was caused by increasing the adhesion forces from the hydrogen bonds formed between the filler and matrix [48].

### 3.9. Antimicrobial Activity

**Figure 8:**
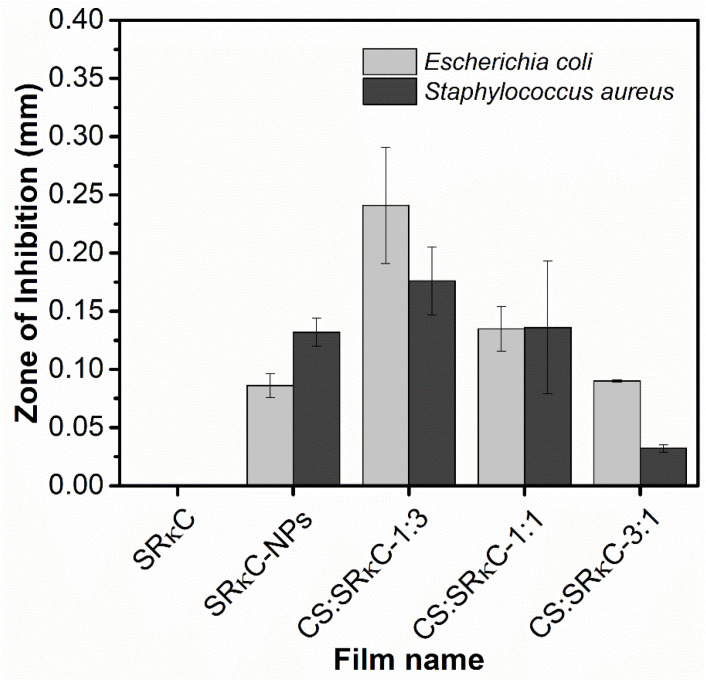
Inhibition zone of as-prepared bionanocomposite films.

The antimicrobial activity of the films indicated microbial growth inhibition due to the formation of the inhibition zone. As shown in Figure 8, the inhibition zone of the SRκC control film did not show antimicrobial activity against both *E. coli* and *S. aureus*. However, the inhibition zone of the SRκC−NP film at various CS concentrations exhibited antimicrobial activity against both bacteria *E. coli* and *S. aureus*. This is in agreement with previous studies that found that antimicrobial activity is increased by incorporating ZnO NPs as a filler into the film matrix [5,30,49]. In addition, a previous study showed that the antimicrobial activity of starch-based film with an antimicrobial agent contributed to extending the shelf life of meat packaging. The antimicrobial agent in the form of antimicrobial ions from nanoparticles such as Zn^2+^, which are released by the ZnO NP content, interacts electrostatically with the negative charge of bacterial membranes [30]. This interaction causes the structure of bacterial cell death on the basis of bacterial membrane damage. Moreover, the CS:SRκC 1:3 film with less CS concentration exhibited greater antimicrobial activity than the other film types, due to the compatibility of the proper CS concentration in the SRκC−NP films, as shown in the SEM and AFM analysis. In contrast, the more CS incorporated into the SRκC-based film (CS:SRκC 1:1 and 3:1), the more significantly the antimicrobial activity was reduced. Hence, the proper mixing ratio of CS and the SRκC film (CS:SRκC 1:3) enhanced the antimicrobial activity of the SRκC-based film.

### 3.10. Degradability

**Figure 9:**
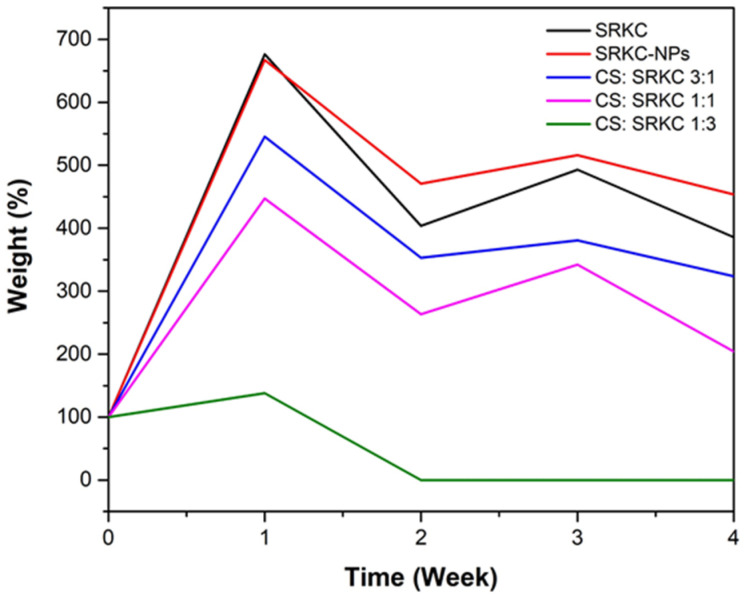
Degradability of bionanocomposite films.

Figure 9 shows the degradation rate of the films in the soil as a weight (%) over a period of 4 weeks, where the weight reduction indicates the degradability of the film. After the first week, the weight of all film types was increased due to the absorption of water by the hydrophilic SRκC films from the moist soil. In contrast, the lowest absorption of water was obtained for the CS:SRκC 1:3 film. The films began to deteriorate the following week, and only the CS:SRκC 1:3 film degraded entirely after two weeks, while the degradation of the other films was generally slower than the CS:SRκC 1:3 film. The enzymatic responses of living organisms (bacteria, yeast, and mold) and the presence of specific enzymes damaged the film. The polymer chain was broken into small fragments that the microorganisms use as energy sources [29]. This result indicates that the incorporation of SiO_2_ and ZnO nanoparticles impedes the biodegradation process of the film, except when the CS-to-SRκC ratio increases. Prevention of the penetration and diffusion of microorganisms into the film matrix was reported to be responsible for impeding the biodegradation process due to the presence of nanoparticles providing a catalyst effect and their large aspect ratio and dispersion in the film matrix providing more tortuous pathways [32]. 

### 3.11. Determination of the Best Film Type

In this study, the best incorporation of CS at various concentrations into the previously developed bionanocomposite film was determined using a nondimensional scaling model, as shown in Table 4. Among the other films, the CS:SRκC 3:1 film exhibited the thinnest film than the other types. Nevertheless, all films with CS addition are considered to be transparent according to the Japanese industrial standard [50]. Hence, according to the parameters of the mechanical properties, wettability, and antimicrobial activity, the CS:SRκC 1:3 film appeared to be the best film type than the other types of film (with a total score of 0.64). CS:SRκC 1:3 with less CS concentration was demonstrated to have the best film properties. Hence, a more detailed investigation of film packaging type was carried out for the CS:SRκC 1:3 film performance compared to the SRκC and SRκC−NP films. In addition, the sample of minced chicken without any film packaging was investigated as the control. The performance parameters of the film packaging were pH, total volatile base nitrogen (TVBN), total plate count (TPC), and thiobarbituric acid-reactive substances (TBARS), which were used as an indicator of chicken deterioration, microbial quality, and malonaldehyde concentration of minced chicken content, respectively.

### 3.12. pH Values

The physical and microbiological quality of the minced chicken sample is indicated by its pH (Table 5). The pH value of the minced chicken sample was increased gradually for 12 days. As shown in Table 5, all pH values of samples were acceptable until 9 days, indicating that the pH value of all film packaging types did not exceed the pH-permitted range of 5.7–7.0 [48,51,52]. As indicated in Table 5, the pH value of all film packaging types increased gradually after 9 days of food storage investigation. An increasing pH value of minced chicken probably occurred due to the alkaline compound from its microbial activity probably accumulating. However, the pH value of the unpacked minced chicken was more alkaline than the sample wrapped with the SRκC, SRκC−NP, and CS:SRκC 1:3 film packaging until 9 days of storage time. The lower pH value of the sample that was packaged in the SRκC−NP and CS:SRκC 1:3 film was caused by the antibacterial activity of the developed film, as previously discussed. The pH value of 5.9–6.3 was obtained after 9 days of observation when the CS:SRκC 1:3 film was applied as minced chicken packaging.

### 3.13. Total Volatile Base Nitrogen (TVBN) 

The acceptability limit of the TVBN value for the minced chicken sample must not exceed 25 mg N/100 g [42]. As shown in Table 5, the SRκC−NP and CS:SRκC 1:3 film packaging samples were acceptable until 9 days of investigation time. However, the TVBN value for the minced chicken without any film packaging exceeded the acceptable TVBN level within 6 days of storage time. Additionally, the TVBN value of the SRκC packaging without CS and NP incorporation exceeded the acceptability limit of TVBN within 6 days of storage time. Moreover, the TVBN value for the sample wrapped with the CS:SRκC 1:3 film was lower than the SRκC–NP film packaging, before 12 days of storage time, because they also exhibited antimicrobial activity against both *E. coli* and *S. aureus*. This result indicates that the incorporation of CS and ZnO−SiO_2_ NPs into the SRκC-based films successfully prevented the minced chicken’s deterioration by microbial activity. Hence, the incorporation of CS and ZnO−SiO_2_ NPs into the SRκC-based film packaging successfully prolonged the minced chicken shelf life up to 3 days longer than the unwrapped sample and SRκC packaging.

### 3.14. Total Plate Count (TPC) 

The acceptability limit of the TPC value for the minced chicken sample is 6.0 log CFU/g [45,46,49]. As shown in Table 5, all film packaging types exhibit a TPC value in the range of 5.5 log CFU/g–7.3 log CFU/g after 12 days of investigation time, except for the unpacked minced chicken sample. After 3 days of storage, the TPC value for the minced chicken sample without any film packaging was unacceptable. This result is in agreement with the pH and TVBN analysis, which show that unpacked minced chicken tends to produce more alkaline conditions and a higher TVBN value due to deterioration by microbial activity. In contrast, the TPC value for the minced chicken sample wrapped with the CS:SRκC 1:3 and SRκC−NP film packaging was acceptable after 6 days of storage time. The incorporation of CS and ZnO−SiO_2_ NPs into the SRκC based film tended to inhibit microbial growth because of the antimicrobial activity of the film. This TPC result was consistent with the pH and TVBN analysis, which indicated that the incorporation of CS and ZnO and SiO_2_ NPs into the SRκC-based film packaging resulted in extending the food’s shelf life for 3 days compared to the unwrapped minced chicken sample.

### 3.15. Thiobarbituric Acid-Reactive Substances (TBARS) 

Table 5 shows the TBARS value, which indicated the food quality and rancidity of all film packaging samples. The acceptability limit of the TBARS value is 0.5 mg MDA/kg for minced chicken [53,54]. As shown in Table 5, the TBARS value for the minced chicken without film packaging exceeded the TBARS limit only on the 1st day of storage time. However, the TBARS value for the minced chicken sample, which was wrapped in the SRκC, SRκC−NP, and CS:SRκC 1:3 film packaging was acceptable for 6 days of storage time. On Day 12 of investigation time, the TBARS value of the CS:SRκC 1:3 film packaging sample was slightly lower than the other sample packaging, which indicated the prevention of lipid oxidation by transparency and the UV barrier properties of the film with CS incorporation and ZnO−SiO_2_ NP inclusion. This TBARS result was consistent with the pH, TVBN, and TPC analysis that the incorporation of CS into the SRκC−NP film packaging successfully extended the food’s shelf life for 6 days longer than the unwrapped minced chicken. Therefore, the CS:SRκC 1:3 film packaging is promising to be applied as a preservative for minced chicken packaging.

### 3.16. Weight Loss (WL)

Table 5 shows the WL value. which was significantly (*p* < 0.05) different between the samples during the 12 days of investigation time. After 12 days of investigation, the highest WL value was shown by the minced chicken sample without film packaging. The high value of WL correlated with the reduced minced chicken quality, which affects customer preferences. In contrast, the SRκC film packaging had the lowest weight loss after 12 days due to the WVP properties of the film, which inhibited water transportation through the film matrix and then reduced the weight loss of the sample. The WL value of the SRκC−NP and CS:SRκC 1:3 film packaging was lower compared to the SRκC film packaging, though it was not statistically significant compared to the SRκC film packaging for 9 days. In contrast, the CS:SRκC 1:3 film packaging showed WL values that were significantly different compared to the control. It was found that the incorporation of ZnO−SiO_2_ NPs and CS in the matrix reduces the density of the SRκC film packaging. A similar trend was reported by Praseptiangga et al. [49]. However, the obtained CS:SRκC 1:3 film packaging was the most transparent among the primary goals of this study by incorporation of CS.

### 3.17. Total Color Difference (∆E)

Table 5 shows the total color difference (∆E), which represents the color analysis of the minced chicken sample. The maximum allowable limit of the ∆E value is less than 4 [55]. Table 5 demonstrates that all film packaging samples were acceptable until 9 days of storage time, indicating that the UV barrier properties of the film prevented the color of the minced chicken from oxidizing.

According to the discussion, the incorporation of CS into the SRκC-based film (CS:SRκC 1:3) provided a proper composition ratio with certain nanoparticle inclusion. The CS:SRκC 1:3 film enhanced the transparency, thickness, tensile strength, and antimicrobial activity against both *E. coli* and *S. aureus*. The CS:SRκC 1:3 film packaging was also demonstrated to have the best film performance as minced chicken packaging according to the food parameters, including pH, TVBN, TPC, TBARS, WL, and total color difference (∆E). Furthermore, the CS:SRκC 1:3 film packaging was successful in extending the shelf life of the minced chicken sample for up to 6 days longer compared to the unwrapped sample. The above results suggest that the CS:SRκC 1:3 film application was promising for use as active packaging for minced chicken.

## 4. Conclusions

The incorporation of cassava starch (CS) into semi-refined kappa carrageenan (SRκC)-based film with nanoparticle inclusion achieved the multifunctional properties of the film. The incorporation of CS at various concentrations into the film enhanced the intermolecular interaction between ZnO−SiO_2_ NP filler. CS incorporation into the SRκC-based film increased the transparency, thickness, and tensile strength properties of the developed film, which is required for application as food packaging. Moreover, CS incorporation into the SRκC-based film improved the film’s surface smoothness, water vapor barrier, and mechanical properties, including tensile strength. CS incorporation into the SRκC-based film with ZnO−SiO_2_ NP inclusion promoted the antimicrobial activity of the SRκC-based film. It was concluded that the best performance of the prepared SRκC-based films with the incorporation of various CS concentrations was the CS:SRκC 1:3 film. The CS:SRκC 1:3 film packaging successfully extended the minced chicken shelf life up to 6 days based on TVBN, TPC, and TBARS analysis. Thus, the incorporation of CS into SRκC-based film with ZnO−SiO_2_ NP inclusion is promising for use as active food packaging film with environmentally friendly materials.

## Figures and Tables

**Table 1:** Composition of bionanocomposite films.

Film Name	ZnO NPs (g)	SiO_2_ NPs (g)	CS (g)	SRκC (g)	Aquadest (g)	Sorbitol (g)	SDS (g)	Total (g)
SRκC	-	-	-	2.0	97.0	1.0	-	100
SRκC−NPs	0.3	0.1	-	2.0	96.6	1.0	0.04	100
CS:SRκC 1:3	0.3	0.1	0.5	1.5	96.6	1.0	0.04	100
CS:SRκC 1:1	0.3	0.1	1.0	1.0	96.6	1.0	0.04	100
CS:SRκC 3:1	0.3	0.1	1.5	0.5	96.6	1.0	0.04	100

**Table 2:** Thickness, tensile strength (TS), elongation at break (EB), and water vapor permeability (WVP) of the bionanocomposite films.

Film Name	Thickness (µm)	Tensile Strength (MPa)	Elongation at Break (%)	WVP (10^−6^ g·h^−1^·m^−1^·Pa^−1^)
SRC	57.30 ± 8.17 ^a^	28.63 ± 0.61 ^a^	2.76 ± 1.22 ^a^	1.05 ± 0.05
SRκC−NPs	58.14 ± 7.85 ^a^	30.86 ± 0.56 ^ab^	4.02 ± 1.00 ^ab^	0.88 ± 0.11
CS:SRκC 1:3	55.70 ± 6.21 ^ab^	32.44 ± 0.14 ^a^	3.39 ± 0.29 ^a^	0.84 ± 0.04
CS:SRκC 1:1	55.10 ± 8.07 ^ab^	17.58 ± 0.30 ^ab^	4.39 ± 0.82 ^ab^	0.84 ± 0.04
CS:SRκC 3:1	53.52 ± 7.33 ^b^	9.4 ± 0.44 ^b^	7.56 ± 0.39 ^b^	0.83 ± 0.08

Values are presented as mean ± deviation standard, different letters in the same column indicate significantly different (*p* < 0.05).

**Table 3:** Transmittance of bionanocomposite films.

Film Name	% Transmittance @ λ = 280	% Transmittance @ λ = 550
SRκC	25.14	62.69
SRκC−NPs	6.34	53.33
CS:SRκC 1:3	8.05	58.26
CS:SRκC 1:1	9.17	61.86
CS:SRκC 3:1	9.72	63.57

**Table 4:** Determination of the best film type using several variables.

Film Name	Transparency	UV Barrier	Tensile Strength	Elongation at Break	WVP	Antimicrobial Activity	Total
SRκC	62.69	25.14	28.63	2.76	1.01	0.00	0.24
SRκC−NPs	53.33	6.34	30.86	4.02	0.88	0.21	0.46
CS:SRκC 1:3	58.26	8.05	32.44	3.39	0.84	0.42	0.64
CS:SRκC 1:1	61.86	9.17	17.58	4.39	0.84	0.27	0.55
CS:SRκC 3:1	63.57	9.72	9.41	7.56	0.83	0.42	0.56

**Table 5:** Physicochemical and microbiological quality of minced chicken meat packaged with various treatments.

Parameter	Day	Unpacked Minced Chicken	SRκC Packaging	SRκC−NP Packaging	CS:SRκC 1:3 Packaging
WL (%)	0	0 ^Aa^	0 ^Aa^	0 ^Aa^	0 ^Aa^
	3	8.06 ± 1.21 ^Bc^	3.41 ± 0.31 ^Bab^	2.19 ± 1.15 ^Ba^	4.69 ± 0.63 ^Bb^
	6	11.52 ± 1.17 ^Cc^	4.01 ± 0.43 ^Ca^	4.16 ± 0.31 ^Ca^	6.25 ± 0.39 ^Cb^
	9	18.19 ± 1.06 ^Dc^	5.69 ± 0.49 ^Da^	6.42 ± 0.8 ^Da^	8.65 ± 1.13 ^Da^
	12	22.31 ± 0.79 ^Ec^	6.55 ± 0.44 ^Ea^	7.5 ± 0.75 ^Da^	10.51 ± 1.62 ^Ec^
pH	0	5.99 ± 0.02 ^Aa^	5.99 ± 0.02 ^Aa^	5.99 ± 0.02 ^Aa^	5.99 ± 0.02 ^Aa^
	3	5.97 ± 0.01 ^Aa^	5.99 ± 0.01 ^Aab^	6.01 ± 0.02 ^Aab^	5.98 ± 0.02 ^Aab^
	6	5.98 ± 0.01 ^Aa^	5.97 ± 0.04 ^Aa^	6.04 ± 0.02 ^Ab^	5.99 ± 0.03 ^Aa^
	9	6.43 ± 0.01 ^Bc^	6.38 ± 0.01 ^Ba^	6.41 ± 0.01 ^Bb^	6.39 ± 0.01 ^Ba^
	12	7.46 ± 0.04 ^Cc^	7.07 ± 0.02 ^Ca^	7.31 ± 0.12 ^Cb^	7.32 ± 0.07 ^Cb^
ΔE	0	-	-	-	-
	3	1.63 ± 0.75 ^Aa^	2.47 ± 0.38 ^Aab^	3.35 ± 0.49 ^Bb^	2.81 ± 1.33 ^Aab^
	6	3.29 ± 1.65 ^Bab^	2.48 ± 0.86 ^Aab^	1.70 ± 0.42 ^Aa^	4.70 ± 2.67 ^Ab^
	9	2.05 ± 0.48 ^ABa^	3.19 ± 2.25 ^Aa^	1.22 ± 0.33 ^Aa^	3.27 ± 1.82 ^Aa^
	12	9.63 ± 0.06 ^Ca^	9.78 ± 1.73 ^Ba^	9.34 ± 1.74 ^Ca^	9.74 ± 0.33 ^Ba^
TBARS (mg MDA/kg sample)	0	0.23 ± 0 ^Aa^	0.23 ± 0 ^Aa^	0.23 ± 0 ^Aa^	0.23 ± 0 ^Aa^
	3	0.72 ± 0.1 ^Bb^	0.42 ± 0.07 ^Ba^	0.35 ± 0.01 ^ABa^	0.35 ± 0.02 ^Aa^
	6	0.77 ± 0.24 ^Bb^	0.39 ± 0.03 ^Ba^	0.47 ± 0.08 ^Ba^	0.58 ± 0.05 ^Bab^
	9	0.8 ± 0.05 ^Ba^	1.02 ± 0.02 ^Cab^	0.82 ± 0.24 ^Ca^	1.15 ± 0.23 ^Cb^
	12	1.17 ± 0.24 ^Ca^	1.12 ± 0.04 ^Da^	1.08 ± 0.03 ^Da^	1.06 ± 0.16 ^Ca^
TVBN (mg N/100 g)	0	5.42 ± 0.06 ^Aa^	5.42 ± 0.06 ^Aa^	5.42 ± 0.06 ^Aa^	5.42 ± 0.06 ^Aa^
	3	5.54 ± 0.11 ^Aa^	10.66 ± 0.04 ^Bb^	10.94 ± 0.09 ^Bc^	11.06 ± 0.13 ^Bc^
	6	11.08 ± 0.1 ^Ba^	11.09 ± 0.09 ^Ca^	11.00 ± 0.16 ^Ba^	10.71 ± 0.42 ^Ba^
	9	31.49 ± 0.57 ^Cc^	29.35 ± 0.4 ^Db^	23.84 ± 0.26 ^Ca^	23.71 ± 1.06 ^Ca^
	12	48.62 ± 0.2 ^Dd^	40.73 ± 0.3 ^Ec^	39.6 ± 0.31 ^Db^	33.47 ± 0.7 ^Da^
TPC (log CFU/g)	0	5.56 ± 0 ^Aa^	5.56 ± 0 ^Aa^	5.56 ± 0 ^Aa^	5.56 ± 0 ^Aa^
	3	6.02 ± 0.07 ^Ab^	5.76 ± 0.1 ^Ba^	5.6 ± 0.03 ^Aa^	5.61 ± 0.12 ^Aa^
	6	6.77 ± 0.63 ^Ba^	6.1 ± 0.02 ^Ba^	6.08 ± 0.03 ^Ca^	6.01 ± 0.01 ^Ba^
	9	6.41 ± 0.12 ^Ba^	6.23 ± 0.04 ^Ca^	6.24 ± 0.08 ^Ca^	6.21 ± 0.01 ^Ca^
	12	8.07 ± 0.09 ^Cb^	7.21 ± 0.02 ^Da^	7.35 ± 0.06 ^Da^	7.33 ± 0.03 ^Da^

Values are presented as mean ± deviation standard. Mean values in the same column with different lowercase letters (a–d) indicate significant differences among formulations within each parameter. Mean values in the same line with different uppercase letters (A–E) indicate significant differences among days within each parameter. TPC: total plate count, WL: weight loss, ∆E: total color difference, TBARS: total barbituric acid-reactive substances, and TVBN: total volatile base nitrogen).
